# Chemical communication is not sufficient to explain reproductive inhibition in the bumblebee *Bombus impatiens*

**DOI:** 10.1098/rsos.160576

**Published:** 2016-10-19

**Authors:** Mario Padilla, Etya Amsalem, Naomi Altman, Abraham Hefetz, Christina M. Grozinger

**Affiliations:** 1 Department of Entomology, Center for Pollinator Research, Center for Chemical Ecology, The Pennsylvania State University, University Park, PA 16802, USA; 2 Department of Statistics, Huck Institutes of Life Sciences, Clinical and Translational Sciences Institute, The Pennsylvania State University, University Park, PA 16802, USA; 3 Department of Zoology, George S. Wise Faculty of Life Sciences, Tel Aviv University, Tel Aviv, Israel

**Keywords:** chemical communication, pheromone, behaviour, altruism, eusocial, division of labour

## Abstract

Reproductive division of labour is a hallmark of eusociality, but disentangling the underlying proximate mechanisms can be challenging. In bumblebees, workers isolated from the queen can activate their ovaries and lay haploid, male eggs. We investigated if volatile, contact, visual or behavioural cues produced by the queen or brood mediate reproductive dominance in *Bombus impatiens.* Exposure to queen-produced volatiles, brood-produced volatiles and direct contact with pupae did not reduce worker ovary activation; only direct contact with the queen could reduce ovary activation. We evaluated behaviour, physiology and gene expression patterns in workers that were reared in chambers with all stages of brood and a free queen, caged queen (where workers could contact the queen, but the queen was unable to initiate interactions) or no queen. Workers housed with a caged queen or no queen fully activated their ovaries, whereas ovary activation in workers housed with a free queen was completely inhibited. The caged queen marginally reduced worker aggression and expression of an aggression-associated gene relative to queenless workers. Thus, queen-initiated behavioural interactions appear necessary to establish reproductive dominance. Queen-produced chemical cues may function secondarily in a context-specific manner to augment behavioural cues, as reliable or honest signal.

## 1. Background

In colonies of eusocial insects, one or a small number of females (queens) dominate the reproduction of the group, whereas the other females (workers) remain (mostly) sterile [1]. Proximate mechanisms that regulate this reproductive dominance include aggression and pheromones [2–4]. It has been hypothesized that aggression originally mediated this conflict in the ancestral state, and the subsequent evolution of queen pheromones allowed for the development of larger, more harmonious social groups [5]. Although it has not been extensively studied, there is some evidence for the transition from aggression to pheromone signalling in social insects [6–9].

In primitively eusocial insects, such as bumblebees, it has been suggested that reproductive division of labour is regulated by a combination of chemical and behavioural cues [10,11]. In *Bombus terrestris* bumblebees, queens dominate reproduction early in the colony cycle, but near the end of the colony cycle workers activate their ovaries and compete with the queen and each other over the production of haploid males (reviewed in [11]). Furthermore, under queenless conditions, workers form a dominance hierarchy [12] where one (or a few) workers exhibit the majority of the aggressive behaviours, have the highest ovarian activation levels [13] and can inhibit reproduction in subordinate workers [14]. The mandibular gland was initially thought to produce a putative pheromone [15,16], but subsequent studies demonstrated that extracts of queen mandibular glands had no inhibitory effect on biosynthesis of the gonadotropin juvenile hormone in workers [17]. Full body queen extracts did decrease juvenile hormone biosynthesis in workers after three days of exposure but did not impact worker aggression, and ovary activation/egg-laying were not assessed [17]. Furthermore, worker reproduction was not inhibited if workers were separated from the queen by a double mesh screen, and thus volatile chemical cues do not function in this system [18]. Non-volatile compounds on the cuticle of queens and fertile workers were suggested to operate as a reliable fertility signal of their presence and reproductive status [19], and chemical cues from the nest wax affected worker reproduction [20].

Recent studies have suggested that the cuticular hydrocarbon pentacosane (C_25_) may play a role in inhibiting worker reproduction in *B. terrestris*, because workers in queenless colonies exposed to pentacosane exhibited significantly higher rates of ‘oocyte resorption’ [21] and had significantly fewer developing oocytes in their ovaries than workers in queenless colonies exposed to solvent controls [22]. However, exposure of queenless *Bombus impatiens* worker groups to pentacosane did not significantly impact ovary activation rates or terminal oocyte sizes after 10 days of exposure [23]. Furthermore, in this study [23], ‘oocyte resorption’ rates were positively correlated with the duration of egg-laying, suggesting this is not an appropriate parameter to quantify reproductive inhibition. Thus, current evidence suggests that queen-produced chemicals alone may only slightly reduce or delay worker reproduction in bumblebees overall. However, pheromone blends are often quite complex and exposure to single chemicals or extracts may not be sufficient to elicit an appropriate behaviour or physiological response.

Previous studies have suggested that *B. impatiens*, a North American bumblebee species, exhibits less aggression than *B. terrestris* in late-stage colonies and in queenless worker groups [24,25]. Comparisons of our behavioural analyses of these two species also support this hypothesis, though without a common garden experiment it is difficult to know if variation in behaviours are due to species-specific differences or differences in environment or observers (see electronic supplementary material, figure S1). Additionally, while ovary activation rates in small queenless worker groups appear to be similar in both species [23,26,27], rates in full-sized late-stage colonies are higher in *B. terrestris* (50–64%, see [27,28]) than in *B. impatiens* (9–11%, see [23,29]). Overall, these studies suggest that *B. impatiens* may rely more heavily on chemical communication to regulate the reproductive division of labour, making comparative studies between *B. impatiens* and *B. terrestris* an especially interesting system in which to investigate the transition from aggression to pheromone signalling.

Here, we investigated the role of different social cues in inhibiting worker reproduction in *B. impatiens*. First, we evaluated the effect of volatile and contact cues from the queen and pupae/brood on worker ovary activation and egg production (experiment 1). These studies demonstrated that direct contact with the queen was necessary to inhibit worker ovary activation. Next, we examined what aspect of ‘direct contact’ was most relevant: the ability of the workers to visually inspect the queen, the ability of the workers to contact the queen with their antennae (and therefore be exposed to possible contact pheromones), or the ability of the queen to freely behaviourally interact with the workers (experiment 2). For this, we reared the groups of *B. impatiens* workers with (i) an uncaged, free queen (ii) a caged queen and (iii) no queen. We examined the effects of these treatments on worker attraction to the queen, aggression among workers, ovary activation and gene expression patterns associated with reproductive dominance.

## 2. Methods

### 2.1. General bumblebee rearing

Colonies of *B. impatiens* were obtained from Koppert Biological Systems (Howell Michigan, USA). Colonies were approximately two weeks old (based on first worker emergence), with less than 30 workers each and all stages of brood. Colonies were maintained in nest-boxes at a constant temperature of 28–30°C and 40–50% humidity, and supplied *ad libitum* with a sugar solution and honeybee-collected pollen. Callow workers (less than 24 h) of approximately the same size were collected from queenright colonies and randomly assigned to different treatments. Callow workers were used, because there is no impact from combining workers from different colonies (they have not yet established colony-specific nest-mate recognition cues), thereby allowing us to increase the sample sizes [8,27]. For experiment 2, all workers were individually tagged to monitor their behaviours. All workers were flash frozen on dry ice after 10 days of treatment and stored at −80°C until dissection.

### 2.2. Assessment of ovary activation and egg-laying

Ovaries were dissected under a stereomicroscope in double distilled water, and the largest terminal oocyte from three ovarioles (two from one ovary and one from the second ovary) was measured with an eyepiece micrometer. The mean of these oocytes for each bee was used as an index of ovary activation [8,13,23]. Newly laid eggs were counted immediately after workers were collected in queenless/caged queen groups, but not in queenright/‘free’ queen worker groups, because it would be impossible to differentiate worker-laid eggs from queen-laid eggs in these groups.

### 2.3. Worker–worker aggression

Discrete aggressive behaviours were recorded in 10 min intervals at approximately three time points (07.00, 11.00 and 15.00) on days 3 and 4 post-group establishment. Observations were performed on days 3 and 4, because aggression in *B. terrestris* workers was found previously to be highest on these days [13]. The behaviours were (i) *attacking,* where one bee physically contacts another bee aggressively, this may result in biting, pushing, dragging, struggling or attempted stinging; (ii) *darting,* where one bee makes a sudden and directed movement towards another bee without making actual contact; and (iii) *humming,* characterized by short bursts of wing vibrations directed at another bee. Behavioural information was collected for each individually tagged worker.

### 2.4. Expression levels of *vitellogenin* and *Krüppel homologue 1*

From each group of workers in experiment 2, trial 1, we selected the most behaviourally dominant worker based on the highest total number of aggressive behaviours she performed over the 60 min of observation time and examined gene expression patterns in the heads of these workers. We homogenized the heads of individual bees and extracted RNA (see electronic supplementary material). We identified the *B. impatiens* homologues for two candidate genes, *vitellogenin* (*vg*) and *Krüppel-homologue 1* (*Kr-h1*), which have previously been shown to vary with reproductive dominance in *B. terrestris* [30,31], and two previously validated housekeeping genes [31,32], *arginine kinase* and *phospholipase A2*, using the NCBI database. Accession numbers, primer sequences and primer efficiencies are presented in the electronic supplementary material, table S1. Details on primer design, RNA extraction, cDNA synthesis and qRT-PCR can be found in the electronic supplementary material.

### 2.5. Experiment 1: effects of volatile chemicals and contact cues from queen and pupae/brood

We evaluated the oocyte size and egg-laying in workers exposed to queen's volatiles from either reduced (three workers and a queen) or full-sized colonies. All workers at the onset of the experiment were callow (less than 24 h), kept in sets of three, randomly selected from full-sized colonies, and were sampled at the age of 10 days.

**Figure 1.**
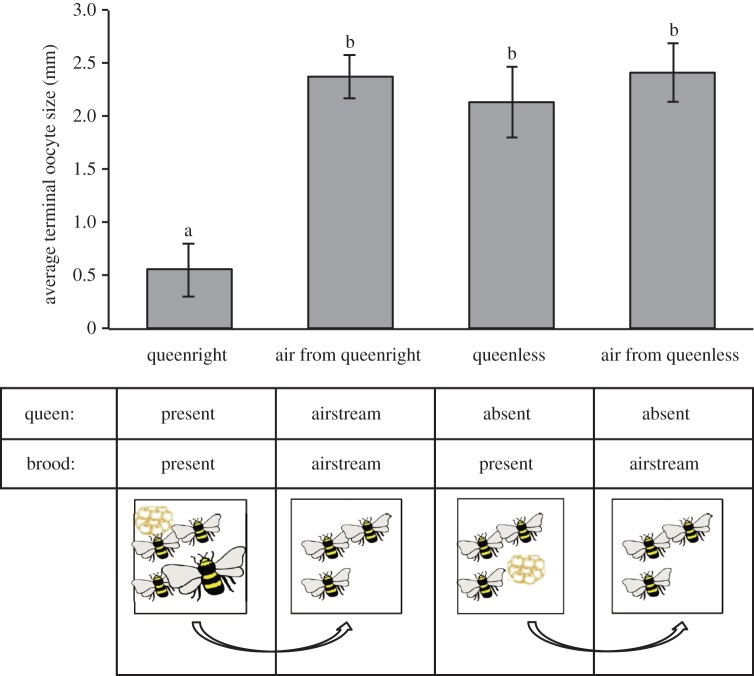
The effect of volatile and contact cues from the queen and brood (pupae) on worker oocyte size. Worker oocytes in reduced colonies (workers + queen + pupae) are significantly smaller than worker oocytes from all other groups, and there are no significant differences observed among the other groups. Thus, only contact with the queen significantly reduces oocyte activation. Note that oocytes are considered ‘ready to lay’ at 2–3 mm [11]. Statistical analyses were performed using a mixed model (*F*_3,20_ = 22.5, *p* < 0.0001 followed by a *post hoc* Tukey test; different letters denote significant differences (*p* < 0.05). Data are presented as means ± standard errors.

A ‘reduced colony’ was created by placing a mated, laying queen from a full-sized colony (*n* = 8, each queen was taken from a different colony, each queen was only used once) into an airtight mason jar (16 oz, wide mouth) with three to five pupae from her colony, along with three callow workers. Reduced colonies were then attached to airtight jars containing three additional callow workers via PVC tubing (VWR, USA). Air was drawn from the callow groups at 200 µl s^−1^ using a laboratory air pump (Gast Manufacturing, Benton Harbor, MI) creating an air flow from the reduced colonies into these groups (figure 1). We employed a double tube system to prevent the buildup of pressure in the jars. As controls, groups of callow workers were exposed to the volatiles from three queenless workers and pupae from the same colony used to generate the ‘reduced colony’. Worker oocyte size was thus measured in 30 groups (workers in reduced colony: *n* = 8 groups; workers exposed to volatiles from reduced colony: *n* = 8 groups; workers with pupae: *n* = 7 groups; workers exposed to volatiles from workers with pupae: *n* = 7 groups). As noted above, the number of eggs laid was measured in queenless groups.

Workers were exposed to full colonies, using the same experimental design as above. Treatment groups containing three callow workers received volatiles from full colonies (queen, more than 30 workers and brood in different stages) housed in brood boxes provided by Koppert (*n* = 12 groups). Control groups received volatiles from an empty brood box (*n* = 12 groups).

### 2.6. Experiment 2: evaluating the effects of visual, contact and behavioural cues from queens

We evaluated the oocyte size, behaviour and gene expression of workers exposed to visual, contact and behavioural cues from the queen. Groups of three callow workers were placed in wooden rearing chambers (21 × 21 × 12 cm) with brood at all stages of development. Workers were either grouped with an uncaged laying queen (free queen), a caged laying queen or left queenless (see below for information on replicates). The queen was caged over the brood and a pollen ball. The mesh cage was small enough that the workers could contact the queen through the mesh easily from all sides, regardless of the position of the queen. Workers were observed to regularly contact the queen through the mesh: we measured worker contact events with the queen during 10 min observation periods every day, and found workers contact the queen an average of 4.38 ± 0.09 times over a 10 min observation period during days 2, 3 and 4 post-worker introduction (data not shown).

We repeated this experiment twice with small modifications. For trial 1, the control queenless worker groups were supplied with an empty cage. In trial 2, to better determine the effect that a moving object within a cage may have, workers were housed with one caged worker. All workers at the onset of the experiment were callows (less than 24 h), randomly selected from full-sized colonies (trials 1: four colonies; trials 2: six colonies), and were sampled at the age of 10 days. Individual workers were also paint-marked to track individuals during behavioural observations.

Worker ovary activation and worker–worker aggression were measured in 19 groups for trial 1 (queenless workers: *n* = 7; caged queen: *n* = 6; uncaged queen: *n* = 6) and 19 groups for trial 2 (queenless workers: *n* = 7; caged queen: *n* = 6; uncaged queen: *n* = 6). Egg number was evaluated only in trial 2. Variation in gene expression levels associated with treatment was evaluated only in trial 1 (queenless workers: *n* = 6; caged queen: *n* = 6; uncaged queen: *n* = 6).

### 2.7. Statistical analyses

The data for all of the experiments are available in the electronic supplementary material, table S2. All statistical analyses were performed using JMP Pro v. 12.1. The oocyte size data were analysed using a mixed model with ‘treatment’ as fixed effect and ‘cage’ as random factor. We also used ‘brood identity’ as random factor but this was not significant (*p* = 0.93, 0.89 for experiment 1 and 2, respectively) and thus was not included in the model. ‘Queen identity’ also did not affect oocyte size (examined using a mixed model with ‘queen identity’ as fixed effect and ‘cage’ as random factor). Multiple comparisons between treatments were performed using a Tukey *post hoc* test. Prior to the analysis, data were log-transformed.

The effect of treatment on egg-laying was examined using a generalized linear mixed model (GLMM) with a Poisson distribution and log as link function (with overdispersion). ‘Queen and brood identity’ were included as random factors. Aggression was analysed using the same GLMM including ‘queen identity’ and ‘cage’ as random factors, with ‘treatment’ and ‘trial’ and the interaction between them as fixed effects. Pairwise comparisons were used in order to compare aggression in the different treatments. Gene expression data were first log-transformed and then analysed using a mixed model with ‘treatment’ as a fixed effect and ‘cage’ as a random factor, followed by a Tukey *post hoc* test. ‘Queen identity’ did not affect gene expression data and thus was not included in the model (examined using a mixed model with ‘queen identity’ as fixed effect and ‘cage’ as a random factor, *p* = 0.44 and 0.68 for *vg* and *kr-h1*, respectively).

Power for the oocyte size and egg count data was computed using R for two-sided tests with no multiple testing adjustment. Results are summarized in the electronic supplementary material, table S3. For oocyte size in experiment 1, power was computed using simulation. For oocyte size in experiment 2, power was based on the ANOVA of log(response). In studies of queen pheromone in honeybees, exposure to a synthetic blend resulted in 50% reduction in the rates of ovary activation [33], whereas studies in a stingless bee species found a greater than 50% decrease in ovary activation rates in workers exposed to a queen extract [34]. For oocyte size, the power to detect a 50% reduction or increase in size was 100% for experiment 1 and 96.2% in experiment 2. For a more modest 25% reduction, power was 60.5% and 43.3%, respectively, but the combined power over the two independent experiments is 77.8%. The power for the egg count data was 86.1% and 10.4%, respectively, for a 50% reduction or increase in count, giving a combined power over the two experiments of 87.5%.

## 3. Results

### 3.1. Experiment 1: effects of volatile chemicals and contact cues from queen and pupae/brood

There was a significant effect of treatment on worker oocyte size in reduced colonies (*F*_3,20_ = 22.5, *p* < 0.0001, figure 1). Workers within the reduced colony (which had direct contact with the queen) had significantly smaller terminal oocytes (0.56 ± 0.25 mm, *p* < 0.001 for all *post hoc* comparisons) than workers exposed to air from reduced colonies (2.37 ± 0.21 mm), workers in queenless groups containing pupae (2.41 ± 0.27 mm) or workers exposed to the air from these groups (2.14 ± 0.33 mm). Thus, in all groups where workers had no direct contact with the queen, worker ovaries were fully activated (*p* > 0.8, ‘ready to lay eggs’ are more than 2 mm), whereas the ovaries of workers that had direct contact with the queen were essentially inactive. Although there was a trend for worker groups exposed to air from reduced colonies to produce fewer eggs (16.4 ± 3.0 eggs, *n* = 7) compared with worker groups exposed to air from workers and pupae (21.6 ± 2.1 eggs, *n* = 8), egg production was highly variable and this difference was not significant (*χ*^2^ = 1.74, d.f.* *= 1, *p* = 0.19).

Next, we repeated the same experiment using three worker groups exposed to volatiles from full colonies (contained a queen and all stages of brood). Terminal oocyte size in workers exposed to volatiles from full colonies did not differ significantly from that of the control group, which consisted of workers exposed to volatiles from an empty rearing chamber, and workers from both treatments had fully activated ovaries (terminal oocyte size of 2.41 ± 0.57 mm versus 2.63 ± 0.087 mm, *F*_1,2.6_ = 0.42, *p* = 0.57). Differences in total eggs laid from these two groups were also not statistically significant (workers exposed to volatiles from full colonies: 18.3 ± 2.3 eggs; workers exposed to volatiles from an empty cage: 22.6 ± 3.3 eggs, *χ*^2^ = 1.20, d.f.* *= 1, *p* = 0.27).

### 3.2. Experiment 2: evaluating the effects of visual, contact and behavioural cues from queens

**Figure 2.**
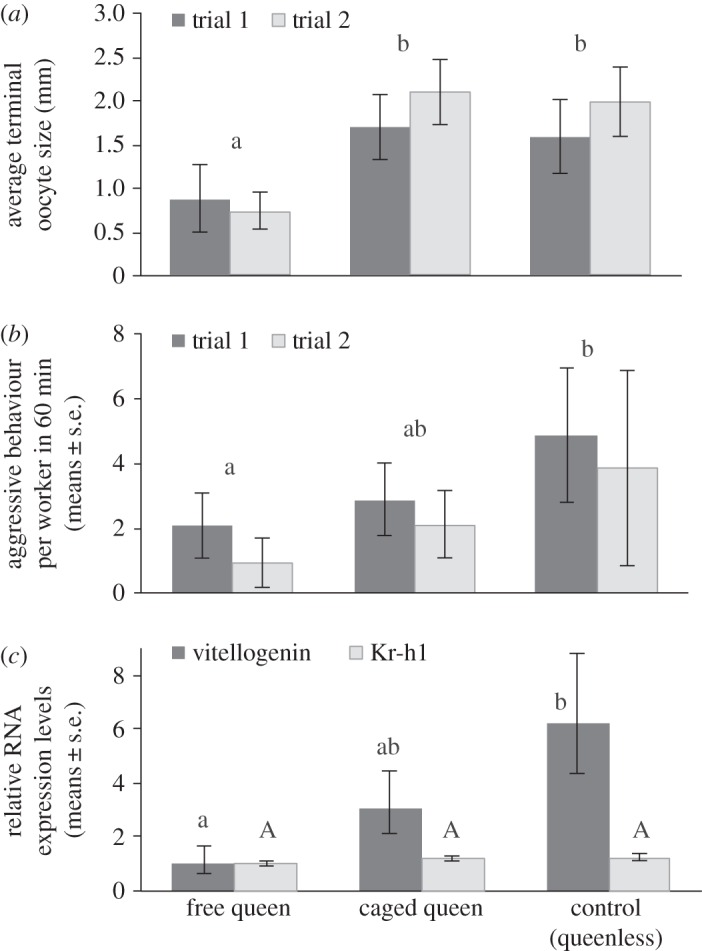
Evaluating the effects of visual, contact and behavioural cues from queens on workers. (*a*) There is a significant effect of treatment on worker oocyte size in both trials. Worker ovary activation is higher in the queenless and caged queen groups relative to the free queen groups, and there is no significant different between queenless workers and workers housed with the caged queen. Different letters denote significant differences (*p* < 0.05). Data are presented as means ± standard errors. (*b*) Aggressive behaviours are significantly higher in queenless worker groups compared with free queen groups, and caged queen groups are intermediate. Additive total of attacking, humming and darting are presented as means ± s.e. Different letters denote significant differences (*p* < 0.05). (*c*) Expression of *vitellogenin* is significantly higher in the heads of behaviourally dominant workers from the queenless controls compared with behaviourally dominant workers in the free queen groups, whereas workers from caged queen groups are intermediate. There is no effect of treatment on *Kr-h1* expression. Different letters denote significant differences (*p* < 0.05); data are presented as means ± s.e.

Worker oocyte size was examined in three treatment groups: (i) three workers grouped with a free, uncaged queen; (ii) three workers grouped with a caged queen; and (iii) three queenless control workers (exposed to an empty cage in trial 1 and to a caged worker in trial 2). All groups contained brood at all stages of development. Worker terminal oocyte size was significantly impacted by treatment (*F*_2,32_ = 8.45, *p* = 0.001), but not by trial (*F*_1,32_ = 1.68, *p* = 0.2) or their interactions (*F*_2,32_ = 0.15, *p* = 0.85; figure 2*a*). Workers housed with free queens had significantly smaller oocytes than workers housed with caged queens or no queens (*p* < 0.012), and there was no difference in oocyte size of workers housed with caged queens or queenless controls (*p* = 0.59). Only workers that had contact with a freely behaving queen had inactive ovaries, whereas workers under queenless conditions or housed with a caged queen had mature, ready-to-lay eggs (terminal oocyte size more than 2 mm) and fully activated ovaries. Note that, because all treatments contained brood at all stages of development, contact with brood was not sufficient to prevent workers from fully activating their ovaries.

There were no significant differences in egg production (measured in trial 2) between workers housed with a caged queen or queenless controls, though again there was a trend for fewer eggs laid by workers housed with a caged queen (queenless control: 10 ± 2.90 eggs, caged queen: 5.67 ± 2.95 eggs, *χ*^2^ = 1.05, d.f.* *= 1, *p* = 0.31).

Next, we evaluated worker–worker aggression among these groups. There were significant differences among the treatment groups (*χ*^2^ = 9.87, d.f.* *= 2, *p* = 0.007, figure 2*b*), but there was no trial effect (*χ*^2^ = 1.92, d.f.* *= 1, *p* = 0.17) or interaction (*χ*^2^ = 0.47, d.f.* *= 2, *p* = 0.79). Aggressive behaviours were significantly higher in queenless worker groups compared with the free queen groups, whereas caged queen groups were intermediate (multiple comparisons: queenless versus free: *p* = 0.002; queenless versus caged queen, *p* = 0.07, caged versus free, *p* = 0.19). Aggression between queens and workers was never observed.

In *B. terrestris*, expression levels of *Krüppel homologue 1* and *vitellogenin* are linked to aggression and dominance. Expression of *Kr-h1* is downregulated in workers in the presence of a queen or a dominant worker [30], and expression of *vitellogenin* is upregulated in aggressive and aggressed workers compared with passive workers [31]. Thus, *Kr-h1* and *vg* are both sensitive to social context, making them appropriate candidate genes to investigate the social cues mediating the reproductive division of labour in bumblebees.

Relative mRNA expression levels of *vg* and *Kr-h1* were analysed for the behaviourally dominant worker (identified based on aggression score) in each cage in each treatment group in trial 1 only. For *vg*, there was a significant effect of treatment (*F*_2,15.5_ = 4.37, *p* = 0.031, figure 2*c*), with expression levels significantly higher in queenless worker groups than free queen groups (*p* = 0.027), whereas caged queen groups were intermediate. There was no significant effect of treatment on *Kr-h1* expression levels (*F*_2,15.5_ = 1.39, *p* = 0.28, figure 2*c*).

## 4. Discussion

Our results demonstrate that reproductive dominance of the *B. impatiens* queen is mediated by behavioural interactions with the queen, which possibly may be reinforced by visual, volatile or contact chemical cues which may serve to place the queen's behaviour in the correct colony context. Interaction with a freely behaving queen is necessary to inhibit ovary activation over a 10 day period: workers exposed to all other treatments had fully activated ovaries with mature, ‘ready-to-lay’ eggs. It is important to note that though our experiments used small groups of three workers, the presence of a freely interacting queen fully inhibited worker ovary activation in these groups, as it would in a full, pre-competition phase colony, and thus the mechanisms needed to reduce worker ovary activation were functional in these small group settings. Workers exposed to a caged queen—and her associated visual, volatile, and contact chemical cues—showed a non-significant trend (relative to queenless workers) of reduction in both aggression at 3–4 days post-group establishment and *vitellogenin* expression levels at 10 days post-group establishment. Furthermore, there was non-significant trend for egg production to be lower in workers exposed to queen volatiles and caged queens relative to queenless controls. Overall, these results suggest that chemical cues from the queen may result in a slight delay in the onset of worker reproduction, but are not sufficient to inhibit it (unlike a freely behaving queen) or even significantly delay it, even over a relatively short timescale of 10 days.

Studies in *B. terrestris* have also suggested that contact with a freely behaving queen is necessary to establish the queen's reproductive dominance in this species, though this can be modulated by colony environment (e.g. in post-competition colonies, workers will activate their ovaries, regardless of their contact with the queen). In *B. terrestris*, queen chemical extracts did not impact aggression [17] and exposure to the hydrocarbon pentacosane (which is found at higher levels in queens than workers, but is still found in workers) did not inhibit worker ovary activation [21], though it was associated with reduced numbers of visible oocytes in the ovaries of exposed workers [22]. Thus, in both *B. impatiens* and *B. terrestris*, queen-produced chemical cues seem to only marginally modulate the timing of worker dominance hierarchy establishment and/or worker egg-laying behaviour when compared with the effects of a freely behaving queen. A previous study in *B. terrestris* [18] used a queen excluder to limit the queen's movement in a confined compartment within the nest while at the same time presenting the workers (which could freely move between compartments) with the opportunity to evade the queen and reproduce. Although worker-laid eggs in the queen-excluded compartment occurred earlier than in the queenright compartment, it was not dramatically earlier, and much later compared with queenless workers. Furthermore, the amount of time the workers spent in the queenright versus queen-excluded compartment was similar and irrespective of ovary activation. These results suggest that the queen's behavioural and chemical cues do not actively and/or negatively inhibit worker ovary activation (if so, the workers would counteract these signals simply by avoiding the queen) but instead the cues allow the workers to adjust the timing of their ovary activation, so they initiate reproduction at the time when it is most beneficial to them. Thus, if any chemical cues produced by the queen operate in bumblebees to regulate worker reproduction, then they are likely to be context-dependent, and to provide workers with information about the queen presence and fecundity rather than to elicit a primer or releaser physiological response in workers. Overall, it suggests that these chemical cues in bumblebees are serving as reliable or honest signals (depending on whether their production is costly) of queen presence and fecundity rather than a pheromonal queen ‘control’ mechanism (see [35] for further discussion). Intriguingly, it may be that chemical signals in the colony serve not to inhibit ovary activation but rather to stimulate it and coordinate its timing with the colony cycle. Indeed, a recent study found that wax scent enables workers to time their reproduction by providing essential information concerning the social condition of the colony: queenright workers exposed to wax from the competition phase were more aggressive and more likely to compete over reproduction than queenright workers without wax or with wax from the early social phase [20].

Previous studies in *B. impatiens* found that aggression and oviposition were less likely to co-occur in workers housed with brood and workers housed without brood tended to be more aggressive and have higher ovarian development, suggesting the presence of a brood pheromone that inhibited worker reproduction [26,27]. In our study, we showed that volatiles from full-sized colonies (which included both queen and brood of all ages), the presence of pupae alone (experiment 1) or physical contact with brood of all ages (experiment 2) did not prevent workers from fully activating their ovaries. The differences between the studies could stem from the differences in the age of the examined workers (12 day old at the onset of the experiment in Sibbald & Plowright [27] and 1 day old in this study) and the aspect of reproduction that was tested (ovarian activation and egg-laying/aggression). Older workers are likely to have activated their ovaries prior to the onset of the experiment (Sibbald and Plowright kept their workers isolated for 12 days prior to pairing them for the experiment) and to modulate their egg-laying in the presence of brood. In accordance with this explanation, studies in *B. terrestris* did not find any effect of brood on the time for workers to initiate their first egg-laying [18] and brood presence did not alter behaviour or worker physiology in queenright groups consisting of 6 day old workers compared with empty-queenright control groups [20].

Interestingly, though vitellogenin is typically described as a ‘yolk protein’, in our studies *vitellogenin* expression closely tracked aggression, but not ovary activation. *Vitellogenin* RNA levels were intermediate in workers housed with a caged queen compared with workers housed with a free queen or no queen, despite the fact that these workers had fully activated ovaries. Previous studies have also found similar results in *B. terrestris* workers [31]: *vitellogenin* RNA levels were not associated with worker task or juvenile hormone titres (but see [36]), and were only partially associated with age, queen presence, caste and ovary activation. However, in *B. terrestris* workers, *vitellogenin* RNA levels were increased in worker bees experiencing high levels of aggression [31]. These results suggest that *vitellogenin* RNA levels in both *B. terrestris* and *B. impatiens* bumblebees are primarily associated with social aggression, though vitellogenin, as a protein, may still play a role in ovary activation. Note that in honeybees (*Apis mellifera*), *vitellogenin* RNA levels are strongly correlated with worker division of labour and juvenile hormone titres [37], though again there is little correlation with queen or worker reproductive state [38], indicating that vitellogenin's functions in social insects are likely much broader than a yolk protein. *Kr-h1* expression levels did not differ significantly between groups, despite the significant differences in ovarian activation and aggression, which is in contrast to studies in *B. terrestris*, where *Kr-h1* expression is reduced in the presence of the queen or dominant workers and positively correlated with juvenile hormone levels [30,36]. However, previous studies evaluated expression in brain and fat body, while we used whole heads, and thus further analysis is necessary to determine if these differences are due to species-level effects or tissue differences.

The factors that inhibit reproduction in workers exposed to a freely behaving *B. impatiens* queen remain to be determined. Aggressive interactions were not observed between workers and queens in our studies, but it may be that more subtle behaviours play a role or low-frequency aggression is sufficient to maintain dominance. It is likely that multiple signals (chemical, visual, behavioural) are used by workers to assess the presence of a fertile queen, with behaviour playing a major role. If the workers do indeed adjust their reproduction according to the cues they obtain from the queen or nest environment, then it will be interesting to determine if there is variation in their likelihood to activate their ovaries and what causes it—in honeybees, for example, worker reproductive potential is negatively correlated with cooperative interactions with the queen [39,40].

Thus, though there is a reduction in worker ovary activation rates in late-stage colonies (see [23,27–29]) and a reduction in aggression during the establishment of reproductive dominance hierarchies (see electronic supplementary material, figure S1 and [24,25]) in *B. impatiens* relative to *B. terrestris*, our results provide no indication that these species-level differences are due to an increased reliance on chemical signalling in *B. impatiens*, and thus some other factors must underpin the observed species-level differences. Indeed, in both species, the presence of an active, live queen that freely interacts with workers appears to be critical for fully inhibiting worker reproduction. Inhibition of worker reproduction appears to be driven primarily by behavioural interactions with the queen, though chemical signals may play a minor role in a context-dependent manner (e.g. they may augment to impact of the behavioural signals obtained from the queen, serve to marginally delay the onset of worker reproduction in the absence of these behavioural signals or allow workers to assess the stage of the colony cycle). However, there are considerable differences in the chemical profiles of sterile and reproductive queens and workers in *B. terrestris* [19,20,31,41] and *B. impatiens* (A.H. 2016, unpublished data), and recent studies demonstrated that chemical cues in the wax of post-competition colonies can actually trigger worker reproduction [20]. Thus, there is substantial chemical complexity in the bumblebee colonies, and it remains to be determined if and how these chemicals function in the social organization of bumblebee colonies.

Across species, the resolution of reproductive conflicts in social Hymenoptera appears to require an interplay between behavioural (e.g. aggression) and chemical signalling. It is generally accepted that in the more primitive clades or in species where colonies have small worker populations, aggression is the primary means for maintaining reproductive skew, but in more advanced species—particularly in species where colonies have a large number of workers—pheromonal signalling has replaced aggression. Although the shift to pheromonal regulation seems adaptive in that it replaces costly behaviour, it is also prone to cheating because the totipotent females have the same genome. Consequently, in most social Hymenoptera, workers can not only activate their ovaries, but also concomitantly start to produce the typical reproductively associated chemical signals or pheromones. Thus, it is maladaptive to totally abandon aggressive behaviour in order to maintain reproductive skew. An example of the tight interplay between aggression and pheromone signalling is demonstrated in the ponerine ant *Dinoponera quadriceps* [42–44]. The gamergate first gains its reproductive dominance by aggression, and after establishing reproductive dominance, these females are typified by producing elevated amounts of 9-hentriacotene. However, this pheromone seems insufficient, because, when challenged, the alpha ant not only aggresses the challenger but also marks her with another pheromone bouquet, emanating from Dufour's gland, which provokes other workers to aggress the challenger. Ants of the genus *Diacamma* possess a pair of thoracic appendages called gemma that produce a pheromone signalling reproductive dominance. Challengers in some of these species are neutralized *a priori*, because upon emergence they are aggressed by nest-mates that mutilate and remove their gemma [45,46]. This physical aggression effectively sterilizes the emerging worker and prevents it from becoming a challenger. Even in societies where the conflict was seemingly evolutionarily resolved (e.g. honeybees, see [47,48]), there is still interplay between pheromone production and aggressive behaviour. When rendered hopelessly queenless, workers start to activate ovaries and concomitantly produce the queen pheromone in their mandibular glands. These workers are recognized by rival workers that aggressively compete with them over reproductive dominance [49]. Therefore, it appears that in the race for reproductive dominance, aggression and chemical signalling are closely intertwined, and their relative importance seems to be idiosyncratic to the species.

## Supplementary Material

Supplementary Figure 1

## Supplementary Material

Supplementary Materials

## Supplementary Material

Supplementary Table 1. Primer sequences

## Supplementary Material

Supplementary Table 2. Complete data set

## Supplementary Material

Supplementary Table 3. Power analysis results

## Data accessibility

All the datasets (oocyte size, egg number, behaviour, gene expression) used in this manuscript are presented in the electronic supplementary material, table S2. The data have also been deposited in Dryad (doi:10.5061/dryad.3p0b5) [50].
