# Supplementary material for: Chemical communication is not sufficient to explain reproductive inhibition in the bumblebee Bombus impatiens
Source: R Soc Open Sci. 2016 Oct 19;3(10):160576. doi: 10.1098/rsos.160576 (PMC5099002; doi:10.1098/rsos.160576)
Supplement: Supplementary Figure 1 [file rsos160576supp1.docx]

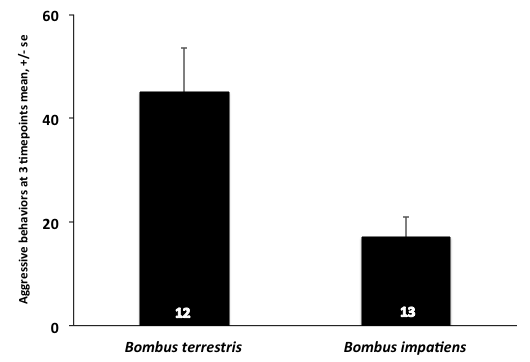


**Supplemental Figure 1. Queenless worker aggression comparison.** Worker aggression data for *B. terrestris* was obtained from Amsalem and Hefetz (2011) and was compared to worker aggression data from this study. Both studies grouped three callow workers together and recorded the same aggressive parameters for a total of 60 minutes on days 3 and 4 post establishment. Aggressive behaviors of *B. terrestris* groups were significantly higher than *B. impatiens* groups (Wilcoxon rank sums; z=2.69, p=0.007). Data are presented as mean +/-SE with the number of replicates presented at the bottom of the bar. It is important to note that the data sets were collected at a different time by different researchers, and thus the results may be influenced by many factors other than the species differences.
