# Supplementary material for: Chemical communication is not sufficient to explain reproductive inhibition in the bumblebee Bombus impatiens
Source: R Soc Open Sci. 2016 Oct 19;3(10):160576. doi: 10.1098/rsos.160576 (PMC5099002; doi:10.1098/rsos.160576)
Supplement: Supplementary Materials [file rsos160576supp2.docx]

**Supplementary Materials**

**Quantitative real-time PCR analysis.** Primers were designed using the Primer3 v 0.4.0 (<http://frodo.wi.mit.edu/>). RNA was extracted from the heads of individual workers using the RNeasy mini kit (Qiagen, Valencia, CA) according to the manufacturer’s instructions. RNA quantity and quality were assayed with a ND-1000 Spectrophotometer (NanoDrop Technologies, Wilmington DE). cDNA was synthesized according to the manufacturer’s instructions using 200 ng of RNA with Reverse Transcriptase (Applied Biosystems, CA, USA).Two microliters of diluted cDNA were combined with 5 µl SYBR-Green (Bioline, Luckenwalde, Germany), 0.2 mM of each gene specific primer and 2.6 µl DEPC-water. Expression levels were determined using quantitative real-time PCR on an ABI Prism®7900 sequence detector with SYBR Green detection method (Applied Biosystems, CA, USA). Triplicate reactions were performed for each of the samples and averaged for use in statistical analysis. Quantification was based on the number of PCR cycles required to cross a threshold of fluorescence intensity (Ct), using the 2^-∆Ct^technique (see Applied Biosystems User Bulletin 2, 'Relative Quantification of Gene Expression'). The geometric mean of the two reference genes was used as a control. Negative control samples (cDNA reaction without RT enzyme) and a water control were also present on each plate. A standard curve was performed for each set of primers using 5 different concentrations of cDNA in order to determine the r^2^ and efficiency.
