# Supplementary material for: Chemical communication is not sufficient to explain reproductive inhibition in the bumblebee Bombus impatiens
Source: R Soc Open Sci. 2016 Oct 19;3(10):160576. doi: 10.1098/rsos.160576 (PMC5099002; doi:10.1098/rsos.160576)
Supplement: Supplementary Table 1. Primer sequences [file rsos160576supp3.docx]

| Gene | Function | Accession number | Forward primer  (5'-3') | Reverse primer  (5' -3') | R^2 | Efficiency |
| --- | --- | --- | --- | --- | --- | --- |
| Phospholipase A2 | Housekeeping | CAY56561 | GGTCACACCGAAACCAGATT | TCGCAACACTTCGTCATTTC | 0.992 | 1.012 |
| Arginine kinase | Housekeeping | AAO40898 | TGTCGGTATCTACGCGCCTG | TTGGTGGATGCTTGTCAGTC | 0.981 | 0.891 |
| Vitellogenin | Reproduction, Aggression | XM_003492229 | CAGCCGCCAATATGATACCT | CCCTCCGTTCGAAGTGATAA | 0.994 | 0.923 |
| Krüppel homolog 1 | Reproduction | XM_003485596 | GAATTGCCAAATCGAGAGGA | GAATTGCCAAATCGAGAGGA | 0.993 | 1.042 |

**Supplementary Table 1. Gene and primer information**
